# Exploring the Impact of Head Group Modifications on the Anticancer Activities of Fatty-Acid-like Platinum(IV) Prodrugs: A Structure–Activity Relationship Study

**DOI:** 10.3390/ijms241713301

**Published:** 2023-08-27

**Authors:** Man Kshetri, Wjdan Jogadi, Suha Alqarni, Payel Datta, May Cheline, Arpit Sharma, Tyler Betters, Deonya Broyles, Yao-Rong Zheng

**Affiliations:** 1 Department of Chemistry and Biochemistry, Kent State University, 236 Integrated Sciences Building, Kent, OH 44242, USAsalqarn3@kent.edu (S.A.); pdatta1@kent.edu (P.D.); mcheline@kent.edu (M.C.);; 2 Department of Chemistry, University of Bisha, Bisha 67714, Saudi Arabia; 3 Department of Chemistry, Case Western Reserve University, Cleveland, OH 44106, USA

**Keywords:** platinum(IV) prodrugs, structure–activity relationship, anticancer

## Abstract

We conducted the first comprehensive investigation on the impact of head group modifications on the anticancer activities of fatty-acid-like Pt(IV) prodrugs (FALPs), which are a class of platinum-based metallodrugs that target mitochondria. We created a small library of FALPs (**1**–**9**) with diverse head group modifications. The outcomes of our study demonstrate that hydrophilic modifications exclusively enhance the potency of these metallodrugs, whereas hydrophobic modifications significantly decrease their cytotoxicity. To further understand this interesting structure–activity relationship, we chose two representative FALPs (compounds **2** and **7**) as model compounds: one (**2**) with a hydrophilic polyethylene glycol (PEG) head group, and the other (**7**) with a hydrophobic hydrocarbon modification of the same molecular weight. Using these FALPs, we conducted a targeted investigation on the mechanism of action. Our study revealed that compound **2**, with hydrophilic modifications, exhibited remarkable penetration into cancer cells and mitochondria, leading to subsequent mitochondrial and DNA damage, and effectively eradicating cancer cells. In contrast, compound **7**, with hydrophobic modifications, displayed a significantly lower uptake and weaker cellular responses. The collective results present a different perspective, indicating that increased hydrophobicity may not necessarily enhance cellular uptake as is conventionally believed. These findings provide valuable new insights into the fundamental principles of developing metallodrugs.

## 1. Introduction

Platinum-based chemotherapy has been a cornerstone of cancer treatment for several decades [1,2]. Key agents such as cisplatin, carboplatin, and oxaliplatin have assumed critical roles in the management of diverse malignancies, encompassing testicular, ovarian, lung, head and neck, and colorectal cancers. These chemotherapeutic agents exert their anticancer effects by instigating the formation of DNA cross-links, which, in turn, impede cancer cell proliferation and elicit apoptosis [1,3,4]. Nonetheless, despite their widespread application, the clinical utilization of platinum-based drugs is encumbered by notable toxicity concerns, culminating in adverse effects such as nephrotoxicity, neurotoxicity, and ototoxicity [1,2]. Furthermore, the regrettably common development of drug resistance and the subsequent cancer relapse in patients underline an imperious necessity to explore novel approaches to platinum-based chemotherapy [5,6,7]. The pursuit of such innovative strategies is envisaged to surmount the prevailing limitations and offer improved therapeutic outcomes for cancer patients [8,9,10,11,12,13,14,15,16,17,18,19,20,21,22,23,24,25,26,27,28,29,30,31,32,33,34,35].

Fatty-acid-like Pt(IV) prodrugs (FALPs) have emerged as a promising new class of Pt-based anticancer agents that utilize innovative drug delivery strategies and cancer biology to overcome the challenges associated with conventional Pt(II) drugs [8,36,37,38,39,40,41]. Designed to mimic the structure of fatty acids, these prodrugs utilize non-covalent interactions with human serum albumin (HSA) for efficient drug delivery [36]. FALPs have demonstrated remarkable stability in whole human blood, reducing their rate of reduction via reducing agents. Furthermore, they possess a distinctive mechanism of action that involves accumulation in mitochondria, inducing mitochondrial damage with the release of Pt(II) payloads, and resulting in increased proapoptotic peroxidase activity and elevated reactive oxygen species (ROS) levels [39]. FALPs have shown potent in vitro activity against a broad range of cancer types and promising in vivo efficacy in various mouse models [42]. Importantly, FALPs can be readily chemically modified to alter their biological activities and chemical properties [37,41,43,44,45,46]. Recent studies have also demonstrated the potential of incorporating these novel Pt(IV) prodrugs into nanoparticles for drug delivery using either non-covalent encapsulation or covalent conjugation based on their amphiphilic structures [37]. Overall, FALPs represent a highly diverse and unique Pt scaffold with promising mechanisms of action that could serve as powerful tools in developing new approaches for cancer therapy. Although the modification of FALPs has predominantly centered around their carboxylic head group, there has not been a comprehensive exploration of the effects of modifying these groups on cellular responses.

**Figure 1:**
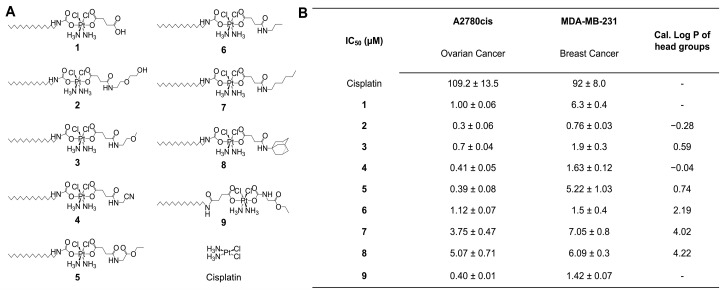
Cytotoxicity profiles of the fatty-acid-like Pt(IV) prodrugs. (**A**) Chemical structures of the Pt(IV) prodrugs (**1**–**9**) and cisplatin. (**B**) Table of IC_50_ values of the Pt compounds against human cancer cells.

**Figure 2:**
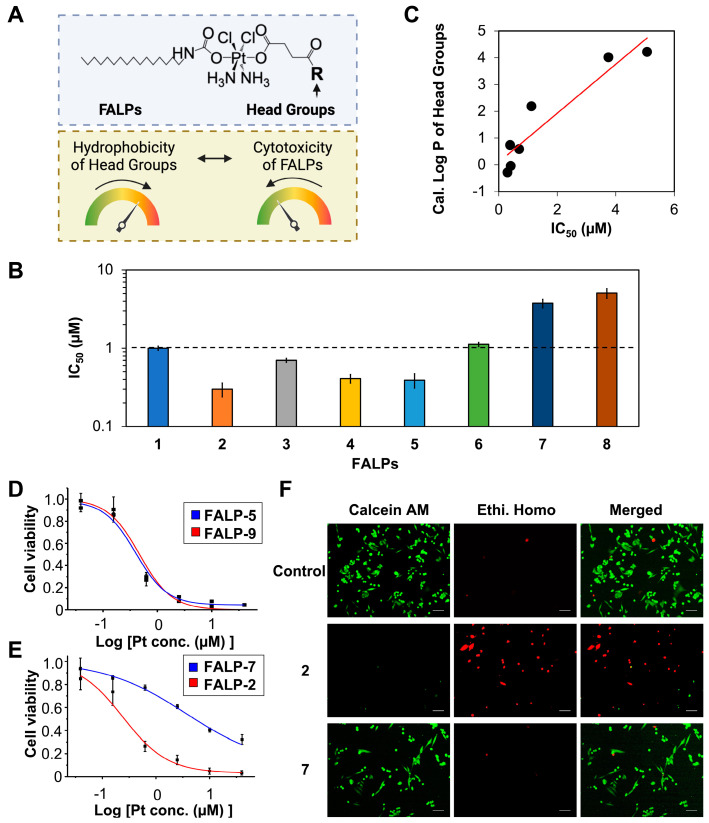
Structure–activity relationship of fatty-acid-like Pt(IV) prodrugs. (**A**) Graphical representation of the hydrophobicity of the head group tuning the cytotoxicity of FALPs. (**B**) A bar graph depicting the IC_50_ values of FALPs (**2**–**8**) with varying levels of hydrophobicity in comparison to unmodified **1** against A2780cis ovarian cancer cells. (**C**) Correlation of the IC_50_ values and the calculated Log P of the head groups of FALPs (**2**–**8**). (**D**) Killing curves of **5** and **9** against A2780cis cells for 24 h. (**E**) Killing curves of **2** and **7** against A2780cis cells for 24 h. (**F**) Live/dead cell assay images of A2780cis cells treated with **2** and **7** ([Pt] = 1 µM) for 24 h. Scale bar = 100 µm.

This new study focuses on exploring the structure–activity relationship of FALP derivatives (**1**–**9** in Figure 1A), with a specific emphasis on how modifications of the carboxylic head group’s hydrophobicity influence their anticancer activity and cellular responses (Figure 2A). Understanding the impact of hydrophobicity on the uptake of therapeutic molecules is widely recognized as a crucial factor in drug development [36,47,48,49,50,51,52,53]. Remarkably, contrary to the widely accepted notion that increased hydrophobicity enhances the cellular uptake of therapeutic molecules, the primary findings of this study demonstrate that FALPs with hydrophilic modifications exhibit exceptional penetration into cancer cells and mitochondria. This, in turn, triggers a cascade of events, leading to substantial mitochondrial and DNA damage, and effectively eradicating cancer cells. On the other hand, increased hydrophobicity in the modifications unexpectedly hinders cellular uptake and mitochondrial accumulation, resulting in weaker cellular responses and a lower in vitro therapeutic efficacy. These findings provide valuable new insights into the fundamental principles of developing metallodrugs.

## 2. Results and Discussion

**Synthesis and characterization of FALP derivatives with various head groups**. The synthesis of the Pt(IV) prodrug (**2**–**8**) is depicted in Figure S1A in the Supplementary Materials. Briefly, amino moieties with different hydrophobicities (**10**–**16**) were conjugated to compound **1** via the HATU-catalyzed amide bond formation reaction. The final compounds were purified via flash column chromatography and/or recrystallization. The overall yields were 44–78%. The synthesis of the Pt(IV) prodrug (**9**) was accomplished in a similar manner, as shown in Figure S1B. The conjugated Pt(IV) compounds (**2**–**9**) were characterized via ^1^H and ^13^C NMR spectroscopy, electrospray ionization mass spectrometry (ESI-MS), and HPLC, and they can be found in the Supplementary Materials (Figures S2–S9). In the ^1^H NMR spectra, the broad signal at ~6.6 ppm corresponds to the amine groups of the Pt(IV) center. The signal at ~2.8 ppm is the CH_2_ group adjacent to the carbamate. The signals at 6.8–8.6 ppm are attributed to the amides in **2**–**8**. In ESI-MS, the isotopically resolved signals agree with the theoretical value of **2**–**9**. The HPLC analysis of the final product indicated that the purity of compounds **2**–**9** from the described synthetic method was >95%.

**Cytotoxicity profiles of FALP derivatives with various head groups** . The in vitro anticancer activity of the Pt(IV) prodrugs (**1**–**9**) was assessed using the 3-(4,5-dimethylthiazol-2-yl)-2,5-diphenyltetrazolium bromide (MTT) assay. This study utilized two human cancer cell lines, namely A2780cis and MDA-MB-231. A2780cis is an ovarian cancer cell line known for its resistance to conventional platinum chemotherapy, making it a formidable challenge to treat. On the other hand, MDA-MB-231 represents a triple-negative breast cancer cell line, which is currently recognized as one of the difficult-to-treat cancer types. The cells were treated with **1**–**9** or cisplatin for 24 h, and the cell viability was evaluated. The IC_50_ values, which represent the concentration of the drug required to inhibit the growth of cells by 50%, are reported in the table in Figure 1B. The results show that **2**–**5** have lower IC_50_ values compared to those of **1** and have lower cisplatin in general. For example, in the A2780cis ovarian cancer cell line, the IC_50_ (**2**) = 0.30 ± 0.06 μM is 36 times lower than that of cisplatin (IC_50_ = 109.2 ± 13.5 μM) and 3 times lower than that of **1** (IC_50_ = 1.00 ± 0.06 μM). Notably, head group modifications do not always increase cytotoxicity more than **FALP-1**. For example, **7** and **8** exhibit much lower cytotoxicity than **1**. Overall, in the A2780cis ovarian cancer cell line, the IC_50_ (**7**) = 3.75 ± 0.47 μM is three times higher than that of **1**, but it is still more potent than cisplatin. Notably, the IC_50_ (**8**) = 5.07 ± 0.71 μM is 15 times higher than that of **2**. Overall, the head group modifications of FALPs result in an alternation of therapeutic effects, which is a promising way to fine-tune the anticancer activity of this class of metallodrugs.

**Structure–activity relationship of FALP derivatives with head groups of different hydrophobicity**. Our goal was to study the relationship between the structure and activity to gain a better understanding of how modifications to the head group affect the anticancer properties of FALP derivatives. We hypothesized that the hydrophobicity of these modifications is a key factor in determining the cytotoxicity of the compounds (Figure 2A). To test this hypothesis, we calculated the Log P values for all the modifications with the ALOGPS 2.1 program, which ranged from −0.28 to 4.22, as shown in Figure 1B. Compounds **2**–**5** had head group modifications with low hydrophobicity (or high hydrophilicity), while compounds **7** and **8** had head groups with high hydrophobicity (or low hydrophilicity). Our results, presented in Figure 2B, indicate that hydrophilic modifications lead to lower IC_50_ values and a higher potency of FALPs, while hydrophobic modifications result in increased IC_50_ values and reduced anticancer activity. To better illustrate the correlation between the hydrophobicity of the head group modifications, we plotted the calculated Log P values against the corresponding IC_50_ values in Figure 2C, which clearly demonstrates the inverse impact of hydrophobicity on the anticancer activities of FALPs in general. Additionally, we sought to determine if this observation was solely based on hydrophobicity, so we engineered two isomers, **5** and **9**. The head group modification of compound **9** was changed from amide to carbamate compared to compound **5**, and interestingly, the cytotoxicity profiles of both **5** and **9** were identical, as shown in Figure 1B and Figure 2D. These results suggest that the hydrophobicity of the structure plays a major role in the structure–activity relationship. Finally, we focused on the two FALPs, **2** and **7**, to illustrate this effect. Although both **2** and **7** were very similar compounds, **2** had a hydrophilic polyethylene glycol (PEG) modification (Log P = −0.28), while compound **7** carried a C6 hydrocarbon chain (Log P = 4.02) of the same molecular weight. As shown in Figure 2E, compound **2** exhibited a much higher potency than **7** in a wide range of concentrations tested, and the IC_50_ (**7**) = 3.75 ± 0.47 μM was 12 times higher than that of **2**. The live/dead cell imaging assays further validated this drastic difference in their in vitro anticancer activity, as shown in Figure 2F, where compound **2** effectively eliminated all drug-resistant A2780cis ovarian cancer cells, while compound **7** was deemed ineffective at the tested concentration ((Pt) = 1 μM). Overall, the combined evidence points out that the hydrophobicity of the head group modifications dictate the anticancer activities of the FALPs.

**Figure 3:**
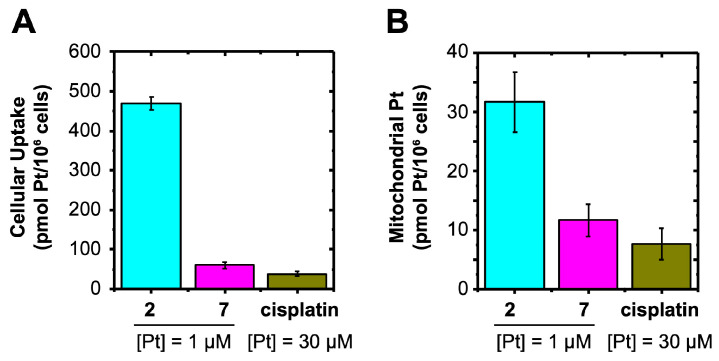
Cellular uptake (**A**) and mitochondrial accumulation (**B**) of FALPs (**2** and **7**) and cisplatin in A2780cis cells (24 h).

**Cell entry and mitochondrial accumulation of FALP derivatives with head groups of different hydrophobicities**. Our next objective was to gain a more detailed understanding of how modifications to the head group of FALPs affect their anticancer activities through hydrophobicity. Cellular uptake is widely recognized as a critical factor in the activity of metallodrugs. The influence of hydrophobicity on the cellular uptake of therapeutic molecules has been widely acknowledged, with an increase in hydrophobicity typically promoting cell entry. To this end, we conducted a graphite furnace atomic absorption spectroscopic (GFAAS) analysis of the cellular uptake for FALPs with various head group modifications, including **2** and **7**. The Log P values of compounds **2** and **7**, as determined via GFAAS, are 1.97 and 2.57, respectively. This indicates that compound **7** exhibits greater hydrophobicity than compound **2**. Surprisingly, our results in Figure 3A indicate that the hydrophilic modification of **2** (496.6 ± 16.09 pmol Pt/million cells) led to an uptake of over eight times greater than the hydrophobic modifications of **7** (60.03 ± 8.01 pmol Pt/million cells). We previously discovered that mitochondria play significant roles in the mechanism of action of FALPs, so we further investigated how the hydrophobicity of the head group modifications affects the mitochondrial accumulation of FALPs. As shown in Figure 3B, the mitochondrial Pt content of **2** (31.7 ± 5.1 pmol Pt/million cells) was three times higher than that of **7** (11.72 ± 2.76 pmol Pt/million cells). Nevertheless, all FALPs demonstrated a higher cellular uptake and mitochondrial accumulation than cisplatin, despite using a higher Pt concentration in the cisplatin sample. In summary, the introduction of a hydrophilic head group in FALPs promotes cell entry and mitochondrial accumulation.

**Figure 4:**
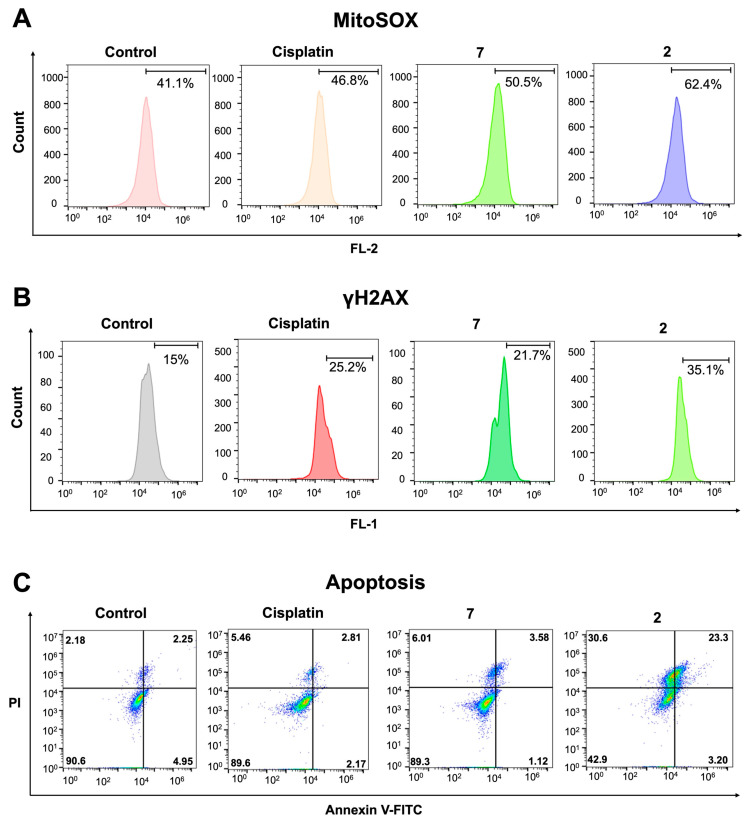
Cellular responses of A2780cis cells treated with FALPs and cisplatin. (**A**) Flow cytometric analysis of MitoSOX in the A2780cis cells treated with FALPs (**2** or **7**) or cisplatin for 24 h. (**B**) Flow cytometric analysis of γH2AX in the A2780cis cells treated with FALPs (**2** or **7**) or cisplatin for 24 h. (**C**) Flow cytometric analysis of apoptosis in the A2780cis cells treated with FALPs (**2** or **7**) or cisplatin for 48 h.

**Cellular Responses of FALP derivatives with head groups of different hydrophobicity**. Based on the observation that hydrophilic head group modifications lead to increased intracellular Pt levels, we formulated the hypothesis that such modifications would result in greater mitochondrial and DNA damage, as well as increased apoptosis. MitoSOX was utilized to assess the levels of mitochondrial ROS, while the γH2AX levels were analyzed to determine DNA damage. As mitochondrial and DNA damage are known to promote apoptosis, Annexin V/PI assays were also conducted to examine the apoptotic effects. A flow cytometric analysis was used to evaluate the mitochondrial ROS, γH2AX, and apoptosis in the cancer cells treated with FALPs (**2** and **7**) in the experiments. As illustrated in Figure 1A, compound **2** has a hydrophilic head group, while compound **7** has a hydrophobic head group. According to the flow cytometric results in Figure 4A, treatment with **2** (1 μM, 24 h) significantly increased the mitochondrial ROS levels compared to the control or **7**. Additionally, the treatment of cisplatin at a higher concentration (10 μM, 24 h) resulted in an insignificant change in the mitochondrial ROS levels, which is consistent with its mechanism of action. The treatment of **2** (0.25 μM, 24 h) also induced DNA damage in the treated A2780cis cells, as shown in Figure 4B. Furthermore, **7** at a higher concentration (1 μM, 24 h) triggered DNA damage, but to a lesser extent than **2**. Likewise, our flow cytometric analysis of Annexin V/PI showed that a larger population of cells were in the late stages of apoptosis (23.3% for **2**) compared to the effect of **7**, which induced only 3.58% of cells to undergo the late stages of apoptosis (Figure 4C). Based on the results, it can be inferred that the FALP derivative (**2**) with a hydrophilic head group modification induces mitochondrial and DNA damage as well as apoptosis more effectively than the one (**7**) with a hydrophobic modification.

## 3. Materials and Methods

**General information**. All reagents were purchased from Strem, Aldrich, or Alfa and used without further purification. Compound **1** was synthesized according to the literature [36]. All reactions were carried out under normal atmospheric conditions. A Bruker 400 NMR was used for NMR data acquisition (frequency: 400 M Hz for ^1^H NMR; 100 MHz for ^13^C NMR). Chemical shifts in ^1^H and ^13^C{^1^H} NMR spectra were internally referenced to solvent signals (^1^H NMR: DMSO at δ = 2.50 ppm; ^13^C NMR: DMSO at δ = 40.45 ppm). The high-resolution mass spectra of created ions were recorded on an Exactive Plus mass spectrometer (Thermo Scientific, Bremen, Germany). Analytical HPLC was conducted on an Agilent 1100 system using C18 reverse-phase columns (Hypersil GOLD; 100 mm × 3 mm; 5 µm). Graphite furnace atomic absorption spectroscopic (GFAAS) measurements were taken on a PinAAcle 900Z spectrometer (PerkinElmer, Shelton, CT, USA). Fluorescence spectra were taken on a FluoroMax-3 Fluorescence spectrophotometer (Horiba, Japan) using the software called FluorEssence. Fluorescence images were acquired using an IX70 (Olympus, Japan) inverted epifluorescence microscope equipped with a digital CCD camera (QImaging, Surrey, BC, Canada). Images were processed, and intensities were quantified with ImageJ software v1.53t. Live/dead cell assay was carried out using Invitrogen (Thermo Fisher Scientific) LIVE/DEAD^TM^ Cell Viability Kit (Cat. No. L3224). Flow cytometry was carried out on a Accuri C6 flow cytometer (Becton, Dickinson and Company Biosciences, Lakes, NJ, USA).

**Synthesis of Compound 2**. An amount of 1 mL of anhydrous DMF was added to compound **1** (70 mg; 0.10 mmol) and HATU (46 mg; 0.12 mmol) in a vial under a stream of Ar and stirred at r.t. for 15 min. 2-(2-aminoethoxy) ethanol (28 µL; 0.28 mmol) was added to the mixture. After 20 min of stirring at r.t., DIPEA (70 µL; 0.41 mmol) was added. The reaction mixture was stirred in the dark at r.t. overnight, centrifuged, and the supernatant was added into 3 mL of brine. Then, the precipitation was collected via centrifugation, washed with water, and lyophilized overnight. Lyophilized product was dissolved in small amount of MeOH and purified with flash chromatography. Yield: 53 mg (67%). ^1^HNMR (400 MHz, DMSO-d_6_): δ: 0.856 (NHCH2(CH2)14C*H3*, t, 3H), 1.23 (NHCH2(C*H2*)14CH3, m, 28H), 2.36 (CO(C*H2*)2CO, m, 4H), 2.88 (NHC*H2*(CH2)14CH3, q, 2H), 3.19 (NHC*H2*CH2O, q, 2H), 3.40 (CH2C*H2*OC*H2*CH2, m, 4H), 3.49 (OCH2C*H2*OH, q, 2H), 6.52 (N*H*CH2(CH2)14CH3, t, 1H), 6.62 (N*H3*, m, 6H), 7.90 (CON*H*(CH2)2O, t, 1H); ^13^C NMR(100 MHz, DMSO-d_6_): δ: 180.4, 172.0, 164.4, 72.6, 69.5, 60.6, 41.4, 40.0, 31.8, 30.3, 29.5, 29.4, 29.2, 27.0, 22.6, 14.4; HR-MS (positive mode) for [C_25_H_54_Cl_2_N_4_O_7_Pt]^+^: *m*/*z* calc: 787.3088, obsd: 787.3064. Purity: 99% determined via HPLC.

**Synthesis of Compound 3**. An amount of 1 mL of anhydrous DMF was added to compound **1** (70 mg; 0.10 mmol) and HATU (46 mg; 0.12 mmol) in a vial under a stream of Ar and stirred at r.t. for 15 min. 2-methoxyethylamine (24 µL; 0.28 mmol) was added to the mixture. After 20 min of stirring at r.t., DIPEA (70 µL; 0.41 mmol) was added. The reaction mixture was stirred in the dark at r.t. overnight, centrifuged, and the supernatant was added into 3 mL of brine. Then, the precipitation was collected via centrifugation, washed with water, and lyophilized overnight. Lyophilized product was dissolved in small amount of MeOH and purified with flash chromatography. Yield: 59 mg (78%). ^1^H NMR (400 MHz, DMSO-d6): δ: 0.836 (NHCH2(CH2)14C*H3*, t, 3H); 1.23 (NHCH2(C*H2*)14CH3, m, 28H); 2.26 (CO(C*H2*)2CO, m, 4H), 2.85 (NHC*H2*(CH2)14CH3, q, 2H); 3.16 (NHC*H2*CH2O, 2H); 3.34 (C*H2*C*H2*OCH3, m, 4H); 3.23 (C*H2*CH2OC*H3*, s, 3H); 6.40 (N*H*CH2(CH2)14CH3 and N*H*3, m, 7H); 7.84 (CON*H*, 1H); 13C NMR (400MHz, DMSO-d_6_): δ: 180.45,171.98, 164.49, 71.09, 58.33, 31.94, 31.73, 30.30, 29.48, 29.12, 26.92, 22.51, 14.36; HR-MS (positive mode) for [C_24_H_52_Cl_2_N_4_O_6_PtH]+: *m*/*z* calc: 758.2986, obsd: 758.2985. Purity: 95% determined via HPLC.

**Synthesis of Compound 4**. An amount of 1 mL of anhydrous DMF was added to compound **1** (70 mg; 0.10 mmol) and HATU (46 mg; 0.12 mmol) in a vial under a stream of Ar and stirred at r.t. for 15 min. To the mixture, 0.5 mL anhydrous DMF solution of aminoacetonitrile bisulfate (43 mg; 0.28 mmol) was added. After 20 min of stirring at r.t., DIPEA (70 µL; 0.41 mmol) was added. The reaction mixture was stirred in the dark at r.t. overnight, centrifuged, and the supernatant was added into 3 mL of brine. Then, the precipitation was collected via centrifugation, washed with water, and lyophilized overnight to afford yellowish-white-colored solid. Yield: 47 mg (65%). ^1^H NMR (400 MHz, DMSO-d_6_): δ: 0.856 (NHCH2(CH2)14C*H3*, t, 3H), 1.23 (NHCH2(C*H2*)14CH3, m, 28H), 2.42 (CO(C*H2*)2CO, m, 4H), 2.87 (NHC*H2*(CH2)14CH3, q, 2H), 4.10 (NHC*H2*CN, d, 2H), 6.51 (N*H*CH2(CH2)14CH3, t, 1H), 6.66 (N*H*3, m, 6H), 8.68 (CON*H*CH2CN, t, 1H); ^13^C NMR (100 MHz, DMSO-d_6_): δ: 180.2, 172.5, 170.5, 118.2, 60.8, 41.1, 31.8, 30.3, 29.5, 29.4, 29.2, 26.9, 22.6, 14.4; HR-MS (positive mode) for [C_23_H_47_Cl_2_N_5_O_5_Pt]^+^: *m*/*z* calc: 739.2677, obsd: 739.2673. Purity: 95% determined via HPLC.

**Synthesis of Compound 5**. An amount of 1 mL of anhydrous DMF was added to compound **1** (70 mg; 0.1 mmol) and HATU (46 mg; 0.12 mmol) in a vial under a stream of Ar and stirred at r.t. for 15 min. To the mixture, 0.5 mL anhydrous DMF solution of glycine ethyl ester hydrochloride (39 mg; 0.28 mmol) was added. After 20 min of stirring at r.t., DIPEA (70 µL; 0.41 mmol) was added. The reaction mixture was stirred in the dark at r.t. overnight, centrifuged, and the supernatant was added into 3 mL of brine. Then, the precipitation was collected via centrifugation, washed with water, and lyophilized overnight to collect yellowish-white-colored solid. Yield: 47 mg (60%). ^1^HNMR (400 MHz, DMSO-d_6_): δ: 0.853 (NHCH2(CH2)14C*H3*, t, 3H, J = 6.9 Hz), 1.15 (COOCH2C*H3*, t, 3H, J = 7.1 Hz), 1.23 (NHCH2(C*H2*)14CH3, m, 28H), 2.39 (CO(C*H2*)2CO, m, 4H), 2.87 (NHC*H2*(CH2)14CH3, q, 2H), 3.792 (NHC*H2*COOCH2, d, 2H), 4.08 (COOC*H2*CH3, q, 2H), 6.50 (N*H*CH2(CH2)14CH3, t, 1H), 6.69 (N*H3*, m, 6H), 8.37 (CON*H*CH2COOCH2, t, 1H); ^13^C NMR(100 MHz, DMSO-d_6_): δ: 180.2, 172.4, 170.4, 60.8, 41.1, 31.6 30.3, 29.5, 29.4, 29.2, 26.9, 22.6, 14.6, 14.4; HR-MS (positive mode) for [C_25_H_52_Cl_2_N_4_O_7_Pt]^+^: *m*/*z* calc: 786.2936, obsd: 786.2933. Purity: 98% determined via HPLC.

**Synthesis of Compound 6**. An amount of 1 mL of anhydrous DMF was added to compound **1** (70 mg; 0.1 mmol) and HATU (46 mg; 0.12 mmol) in a vial under a stream of Ar and stirred at r.t. for 15 min. To the mixture, 0.5 mL anhydrous DMF solution of propylamine (23 µL; 0.28 mmol) was added. After 20 min of stirring at r.t., DIPEA (70 µL; 0.41 mmol) was added. The reaction mixture was stirred in the dark at r.t. overnight, centrifuged, and the supernatant was added into 3 mL of brine. Then, the precipitation was collected via centrifugation, washed with water, and lyophilized overnight to collect yellowish-white-colored solid. Lyophilized product was dissolved in small amount of MeOH and purified with flash chromatography. Yield: 53 mg (72%). ^1^H NMR (400 MHz, DMSO-d_6_): δ: 0.814 (NHCH2(CH2)14C*H3* and NHCH2CH2C*H3* m, 6H); 1.23 (NHCH2(C*H2*)14CH3 and NHCH2C*H2*CH3 m, 28H); 2.25 (CO(C*H2*)2CO, m, 4H, J = 7.1, 42.4 Hz), 2.87 (NHC*H2*(CH2)14CH3, q, 2H); 6.34 (N*H* and N*H3*, 7H); 7.73 (N*H*, s, 1H); ^13^C NMR (100 MHz, DMSO-*d_6_*): δ: 180.54,171.68, 164.46, 32.03, 31.73, 30.30, 29.49, 29.44, 29.35, 29.12, 26.93, 22.80, 14.37; HR-MS (positive mode) for [C_24_H_52_Cl_2_N_4_O_5_PtH]^+^: *m*/*z* calc: 742.3037, obsd: 742.3034. Purity: 95% determined via HPLC.

**Synthesis of Compound 7**. An amount of 1 mL of anhydrous DMF was added to compound **1** (70 mg; 0.1 mmol) and HATU (46 mg; 0.12 mmol) in a vial under a stream of Ar and stirred at r.t. for 15 min. To the mixture, 0.5 mL anhydrous DMF solution of hexylamine (37 µL; 0.28 mmol) was added. After 20 min of stirring at r.t., DIPEA (70 µL; 0.41 mmol) was added. The reaction mixture was stirred in the dark at r.t. overnight, centrifuged, and the supernatant was added into 3 mL of brine. Then, the precipitation was collected via centrifugation, washed with water, and lyophilized overnight to collect yellowish-white-colored solid. Lyophilized product was dissolved in small amount of MeOH and purified with flash chromatography. Yield: 54 mg (69%). ^1^H NMR (400 MHz, DMSO-d_6_): δ: 0.83 (NHCH2(CH2)14C*H3* and NHCH2(CH2)4C*H3*, 6H); 1.23 (NHCH2(C*H2*)14CH3 and NHCH2(C*H2*)4CH3, 36H); 2.24 (CO(C*H2*)2CO, m, 4H), 2.87 (NHC*H2*(CH2)14CH3, q, 2H); 2.97 (NHC*H2*(CH2)4CH3, 4H); 6.48 (N*H* and N*H3*, 7H); 7.79 (N*H*, 1H); ^13^C NMR (100 MHz, DMSO-*d_6_*): δ: 180.47,171.60, 164.41, 31.99, 31.75, 31.46, 29.51, 29.16, 26.94, 26.57, 22.55, 22.52, 14.42; HR-MS (positive mode) for [C_27_H_58_Cl_2_N_4_O_5_PtH]^+^: *m*/*z* calc: 784.3507, obsd: 784.3504. Purity: 95% determined via HPLC.

**Synthesis of Compound 8**. An amount of 1 mL of anhydrous DMF was added to compound **1** (70 mg; 0.1 mmol) and HATU (46 mg; 0.12 mmol) in a vial under a stream of Ar and stirred at r.t. for 15 min to obtain pale-yellow-colored solution. To the mixture, 0.5 mL anhydrous DMF solution of 1-adamantylamine (42 mg; 0.28 mmol) was added. After 20 min of stirring at R.T., DIPEA (70 µL; 0.41 mmol) was added. The reaction mixture was stirred in the dark at r.t. overnight. The solution turned into a golden yellow color. It was centrifuged, and the supernatant was added into 3 mL of brine. Then, the precipitation was collected via centrifugation, washed with water, and lyophilized overnight. Lyophilized product was dissolved in small amount of MeOH and purified with flash chromatography. Yield: 56 mg (67%). ^1^H NMR (400 MHz, DMSO-d_6_): δ: 0.858 (NHCH2(CH2)14C*H3*, t, 3H 1.23 (NHCH2(C*H2*)14CH3, m, 28H), 1.60 (CHC*H2*CH, Adamantyl, t, 6H), 1.90 (CC*H2*CH, Adamantyl, d, 6H), 1.98 (CH2C*H*(CH2)2, Adamantyl, m, 3H), 2.31 (CO(C*H2*)2CO, m, 4H), 2.87 (NHC*H2*(CH2)14CH3, q, 2H), 6.52 (N*H*CH2(CH2)14CH3, t, 1H), 6.66 (N*H*3, m, 6H), 7.30 (CON*H*C(CH2)3, s, 1H); ^13^C NMR(100 MHz, DMSO-d_6_): δ: 180.7, 171.2, 164.4, 51.0, 41.5, 41.4, 36.6, 31.8, 29.5, 29.4, 29.3, 26.9, 22.6, 14.4; HR-MS (positive mode) for [C_31_H_60_Cl_2_N_4_O_5_Pt]^+^: *m*/*z* calc: 834.3664, obsd: 834.3660. Purity: 96% determined via HPLC.

**Synthesis of Compound 9**. To PtC16 (80 mg; 0.122 mmol) and ethyl isocyanatoacetate (16 µL; 0.14 mmol) in a vial, 1.5 mL of anhydrous DMF was added under a stream of Ar and stirred at r.t. overnight. The product was extracted with Et_2_O, washed with H_2_O, and lyophilized overnight to obtain a yellowish-white-colored solid. Yield: 42 mg (44%). ^1^HNMR (400 MHz, DMSO-d_6_): δ: 0.854 (NHCH2(CH2)14C*H3*, t, 3H), 1.18 (COOCH2C*H3*, t, 3H), 1.23 (NHCH2(C*H2*)14CH3, m, 28H), 2.34 (CO(C*H2*)2CO, m, 4H), 2.97 (NHC*H2*(CH2)14CH3, m, 2H), 3.72 (NHC*H2*COOCH2, d, 2H), 4.06 (COOC*H2*CH3, q, 2H), 6.647 (N*H3*, m, 6H), 6.83 (N*H*CH2(CH2)14CH3, t, 1H), 7.85 (CON*H*CH2COOCH2, t, 1H); ^13^C NMR(100 MHz, DMSO-d_6_): δ: 180.4, 171.7, 171.6, 158.4, 60.6, 41.9, 40.0, 31.9, 31.8, 30.4, 29.5, 29.3, 29.2, 27.0, 22.6, 14.6, 14.4; HR-MS (positive mode) for [C_25_H_52_Cl_2_N_4_O_7_Pt]^+^: *m*/*z* calc: 786.2936, obsd: 786.2933. Purity: 95% determined via HPLC.

**GFAAS analysis of Log P values for 2 and 7**. The samples were first dissolved with DMSO to create 200 µM stocks. From these stocks, 50 µL was added to a H_2_O:Octanol mixture with a 1:1 volume ratio. This mixture was vortexed for 5 min and subsequently centrifuged for 3 min at 3000 rpm. Following centrifugation, the H_2_O and octanol layers were isolated for analysis. The Pt content in each phase was quantified using GFAAS to calculate the Log P value.

**Cell culture**. A2780cis cell lines were purchased from Sigma-Aldrich and cultured in RPMI 1640 with L-glutamine (Corning, New York, NY, USA) supplemented with 10% FBS (Atlanta Biologicals, USA) and 1% penicillin-streptomycin (Corning). The MDA-MB-231 cell line was obtained via American Type Culture Collection, and cultured in DMEM 1 g/L glucose, with L-glutamine and sodium pyruvate (Corning) supplemented with 10% FBS and 1% penicillin-streptomycin (Corning). All cell lines were cultured at 37 °C under an atmosphere containing 5% CO_2_. Cells were passaged upon reaching 80–90% confluence via trypsinization and split in a 1:5 ratio.

**MTT assays**. Cytotoxicity profiles of compounds **1**–**9** and cisplatin against different cell lines (A2780cis and MDA-MB-231) were evaluated using the MTT assays. A volume of 100 μL of a RPMI or DMEM containing 8 × 10^4^ cells/mL was seeded in 96-well plates. The plates were incubated for 24 h at 37 °C with 5% CO_2_ to allow for adherence of cells. A volume of 50 μL of RPMI or DMEM with various concentrations of cisplatin or compounds **1**–**9** were added to each well of the microplates. The Pt concentrations were determined via GFAAS. After 24 h, a volume of 30 μL of MTT (5.0 mg/mL in PBS, Alfa Aesar, Haverhill, MA, USA) was added to each well of the microplates. After 24 h, the medium was aspirated, and 200 μL of DMSO was added to each well. The plates were shaken gently on a shaker at r.t. for 10 min. Then, the absorbance of purple formazan was recorded at 562 nm with a BioTek ELx800 plate reader. IC_50_ values were determined using Origin software v7.0. All experiments were performed in triplicate.

**LIVE/DEAD cell viability assays**. A2780cis cells were cultured in imaging disks (MatTek, Ashland, MA, USA) at a concentration of 5 × 10^4^ cells with 2 mL of complete medium and incubated for 24 h at 37 °C with 5% CO_2_. The cells were then treated with compound **2 or 7** ((Pt) = 1 μM) and incubated for 24 h at 37 °C with 5% CO_2_. Before the assay, the cells were washed with 1 mL PBS and 1 mL dye-free RPMI to remove serum esterase activity that is generally present in serum-supplemented growth media. A 100 μL volume of LIVE/DEAD working solution (formed by mixing 2 µM of calcein AM and 2 μM ethidium homodimer-1 in PBS) was carefully added to the disk, which was then incubated at r.t. for 30 min. Images were acquired using an Olympus IX70 inverted epifluorescence microscope equipped with a digital CCD camera (QImaging, Surrey, BC, Canada). Images were processed, and intensities were quantified with ImageJ software (NIH).

**GFAAS analysis of cellular platinum contents in A2780cis cells**. A2780cis cells were seeded in a 6-well plate at a concentration of 5 × 10^5^ cells per well and incubated at 37 °C with 5% CO_2_ overnight. Next day, the cells were treated with compound **2** or **7** ((Pt) = 1 μM) or cisplatin ((Pt) = 30 μM) for 24 h at 37 °C with 5% CO_2_. The remaining live cells were harvested via trypsinization and counted. The cells were then digested in 200 μL 65% HNO_3_ at r.t. overnight. The Pt contents in the cells were analyzed via GFAAS. All experiments were performed in triplicate.

**Measurements of mitochondrial platinum contents in A2780cis cells**. A2780cis cells were seeded on a 6-well plate and incubated at 37 °C with 5% CO_2_ overnight. The cells were treated with cisplatin ((Pt) = 30 μM) or compound **2** or **7** ((Pt) = 1 μM) for 24 h at 37 °C with 5% CO_2_. Next, the wells were washed with PBS (1 mL) and harvested via trypsinization (1 mL) and counted. Mitochondrial fractions were isolated using the Thermo Scientific™ Mitochondria Isolation Kit for Mammalian Cells. The mitochondrial fraction was then dissolved in 200 µL 65% nitric acid and shaken at 400 rpm on an Eppendorf ThermoMixer™ F1.5 at r.t. overnight. Next, the fractions were diluted 4× in water and the platinum content was analyzed using GFAAS. All experiments were performed in triplicate.

**Flow cytometric analysis of MitoSOX**. A2780cis cells were seeded in 6-well plate at a concentration of 6 × 10^4^ cells/mL and incubated overnight. Then, the cells were treated with cisplatin ((Pt) = 10 μM) or compound **2** or **7** ((Pt) = 1 μM) and incubated overnight. The medium was aspirated, and cells were washed with 1 mL PBS. Next, the cells were incubated with 5 μM MitoSOX reagent in fresh medium for 60 min at 37 °C with 5% CO_2_ in the dark. Cells were trypsinized and collected. The cell pellet was washed 2 times with PBS. The cells were then re-suspended in PBS with 0.5% BSA to reach 10^6^ cells/mL and analyzed with BD Accuri C6 flow cytometer using FL-2 channel, and data were processed with FlowJo v10.

**Flow cytometric analysis of γH2AX**. A2780cis cells were seeded in a 6-well plate at a concentration of 4 × 10^5^ cells/well. Cells were then incubated at 37 °C with 5% CO_2_ for 24 h. Next, the cells were treated with compound **2** ((Pt) = 0.25 μM), **7** ((Pt) = 1 μM) or cisplatin ((Pt) = 30 μM) and incubated for 24 h. Live cells were collected and 250 μL BD Permeabilization solution was added to re-suspend the cells, which were then incubated for 20 min at 4 °C. Cell pellets were collected, washed twice with 1X BD Perm/Wash buffer, and resuspended in 50 µL of buffer. Alexa 488-anti γH2AX antibody solution was then added, and the samples were incubated in the dark for 60 min at r.t. The final cell pellets were suspended in 500 μL of PBS with 0.5% BSA and analyzed with BD Accuri C6 flow cytometer using FL-1 channel, and data were processed with FlowJo.

**Flow cytometric analysis of apoptosis**. A2780cis cells were seeded in a 6-well plate at a concentration of 3 × 10^5^ cells/well. Cells were then incubated at 37 °C 5% CO_2_ for 24 h. Next, compound **2** or **7** ((Pt) = 0.5 μM) or cisplatin ((Pt) = 7 μM) was added and incubated for 48 h. Both live and dead cells were collected, resuspended in 1mL PBS, and counted. A 1X binding buffer from the FITC Annexin V Apoptosis Detection Kit 1 (BD Biosciences, Franklin Lakes, NJ, USA) was then added to reach a concentration of 10^6^ cells/mL. An amount of 100 µL cell solution was transferred to a fresh 2 mL Eppendorf tube, and 5 µL of both Annexin V-FITC and PI solutions were added to cells. Cells were incubated for 15 min at r.t. in the dark and then brought to 400 µL volume by adding required volume of binding 1X buffer. Cells were then analyzed with FL-1 and FL-3 channels on a BD Accuri C6 flow cytometer and data were processed with FlowJo.

## 4. Conclusions

Our study represents the first comprehensive investigation of the structure–activity relationship of FALPs. We synthesized a small library of FALPs with diverse head group modifications and found that such modifications can greatly affect the cytotoxicity profiles of FALPs, ranging from low to highly potent. Interestingly, a further analysis revealed that only hydrophilic modifications led to a high potency, while hydrophobic moieties resulted in a much lower cytotoxicity. To explore the impact of hydrophobicity on the cytotoxicity of FALPs, we focused on two similar FALPs, one with a hydrophilic PEG head group and the other with a hydrophobic hydrocarbon modification of the same molecular weight. Using these model compounds, we evaluated cellular uptake and mitochondrial accumulation through GFAAS, as well as mitochondrial and DNA damage and apoptosis through flow cytometry. Our comprehensive findings reveal that FALPs incorporating hydrophilic modifications can readily penetrate cancer cells and mitochondria, initiating subsequent cellular responses that effectively eradicate cancer cells. Conversely, FALPs with hydrophobic modifications showed a notably lower uptake and weaker cellular responses. These combined results present an alternative perspective, differing from the conventional belief that increased hydrophobicity invariably enhances cellular uptake. These findings provide valuable new insights into the fundamental principles of developing metallodrugs. It underscores the significance of developing FALPs with hydrophilic modifications, which hold the potential to yield more potent and effective anticancer agents. This study lays the groundwork for future research endeavors aimed at optimizing the structural design of FALPs, with the objective of enhancing anticancer activity while minimizing side effects.

## Data Availability

Not applicable.

## Supplementary Materials

The synthetic schemes and characterization of FALPs can be downloaded at https://www.mdpi.com/article/10.3390/ijms241713301/s1.
