# Supplementary material for: Exploring the Impact of Head Group Modifications on the Anticancer Activities of Fatty-Acid-like Platinum(IV) Prodrugs: A Structure–Activity Relationship Study
Source: Int J Mol Sci. 2023 Aug 27;24(17):13301. doi: 10.3390/ijms241713301 (PMC10487970; doi:10.3390/ijms241713301)
Supplement: Supplementary file 1 [file ijms-24-13301-s001.zip › ijms-2559666-supplementary.pdf]

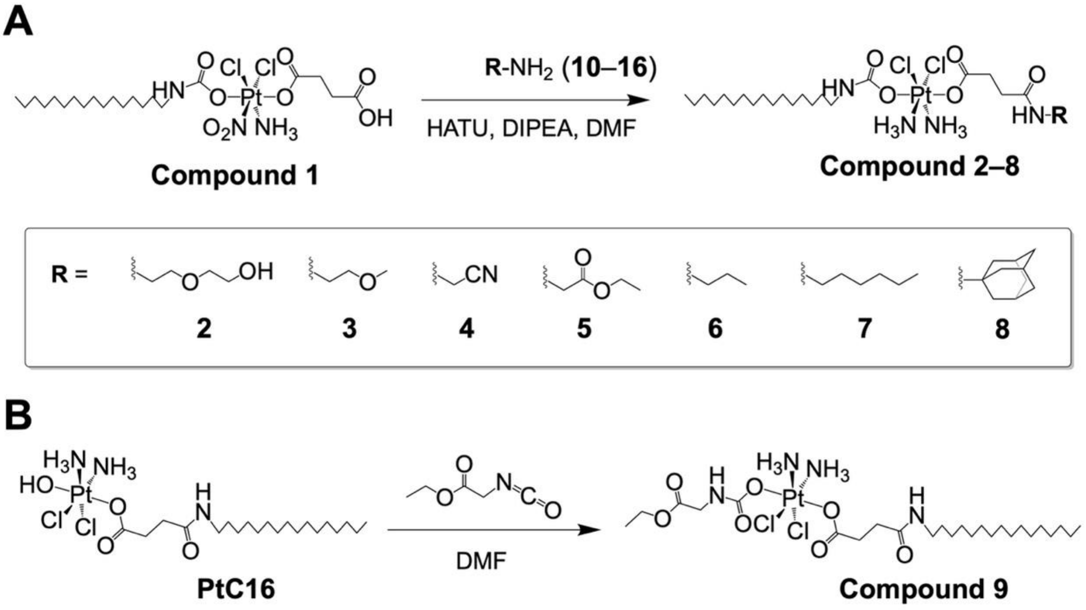

Figure S1. Synthetic routes for preparing FALPs: A. Synthesis of Compound **2–8**; B. Synthesis of Compound **9**.

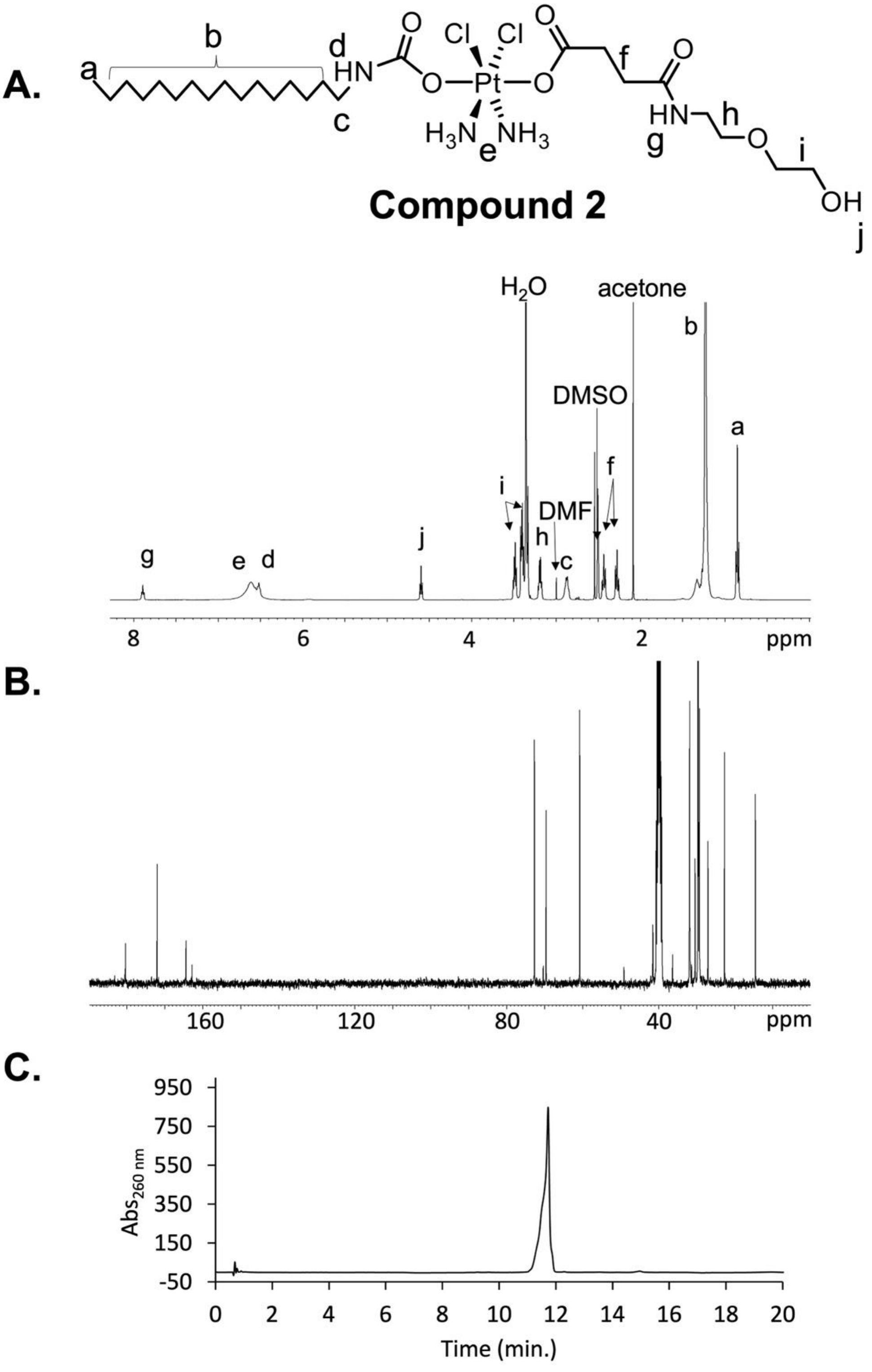

Figure S2. Characterization of Compound **2**: A. ^{1}H NMR; B. ^{13}C NMR; C. HPLC.

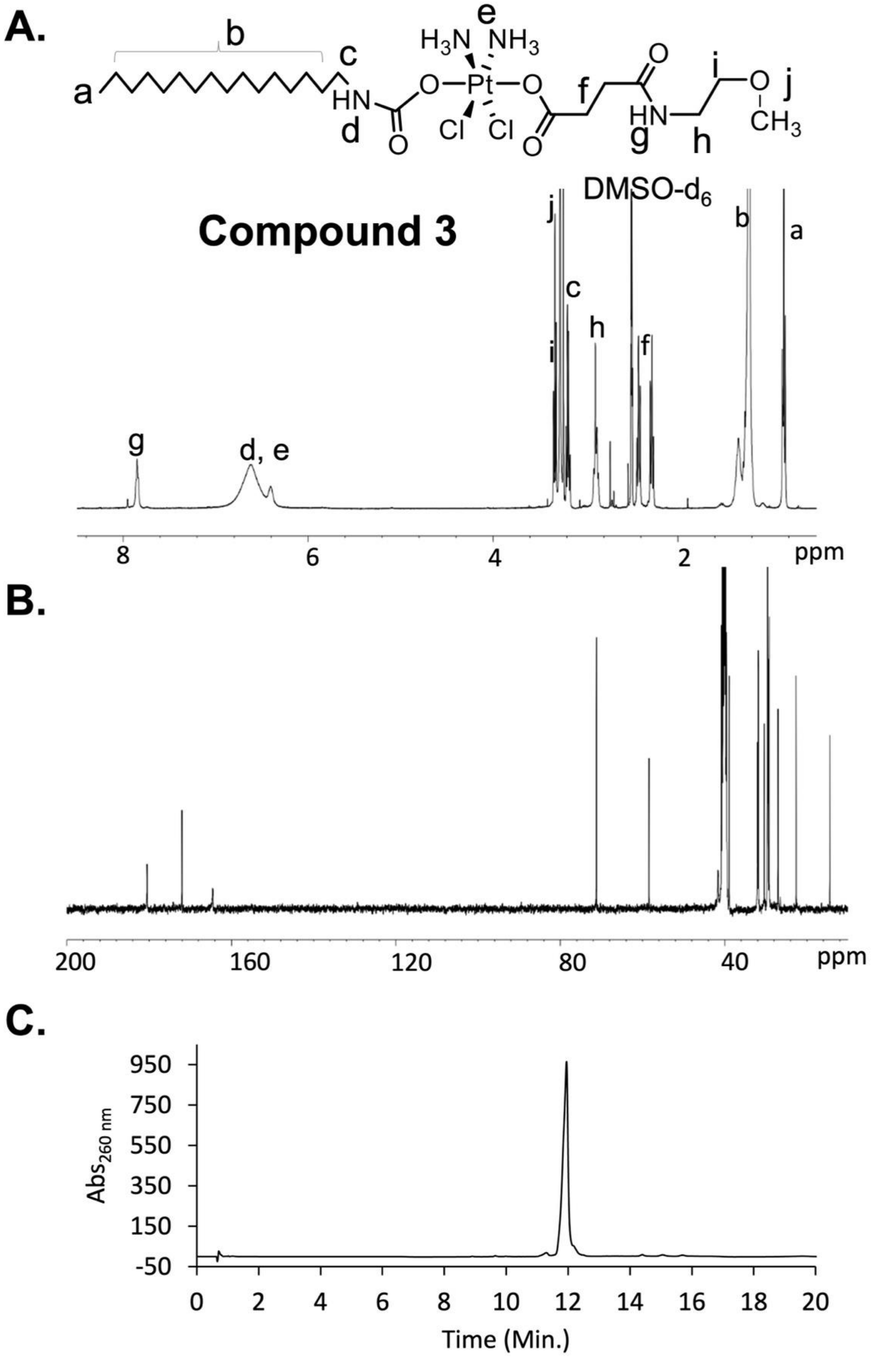

Figure S3. Characterization of Compound **3**: A. ^{1}H NMR; B. ^{13}C NMR; C. HPLC.

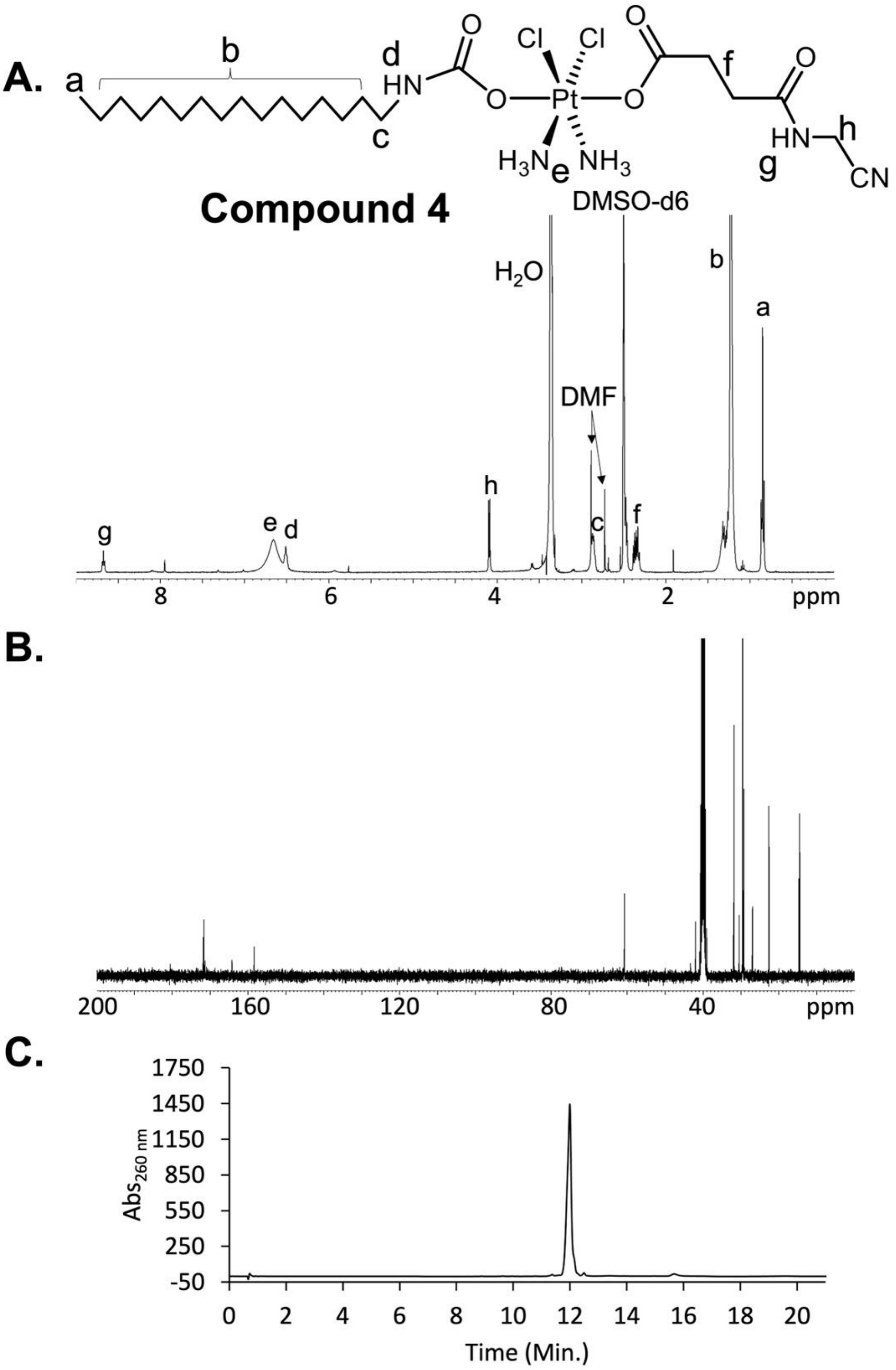

Figure S4. Characterization of Compound **4**: A. ^{1}H NMR; B. ^{13}C NMR; C. HPLC.

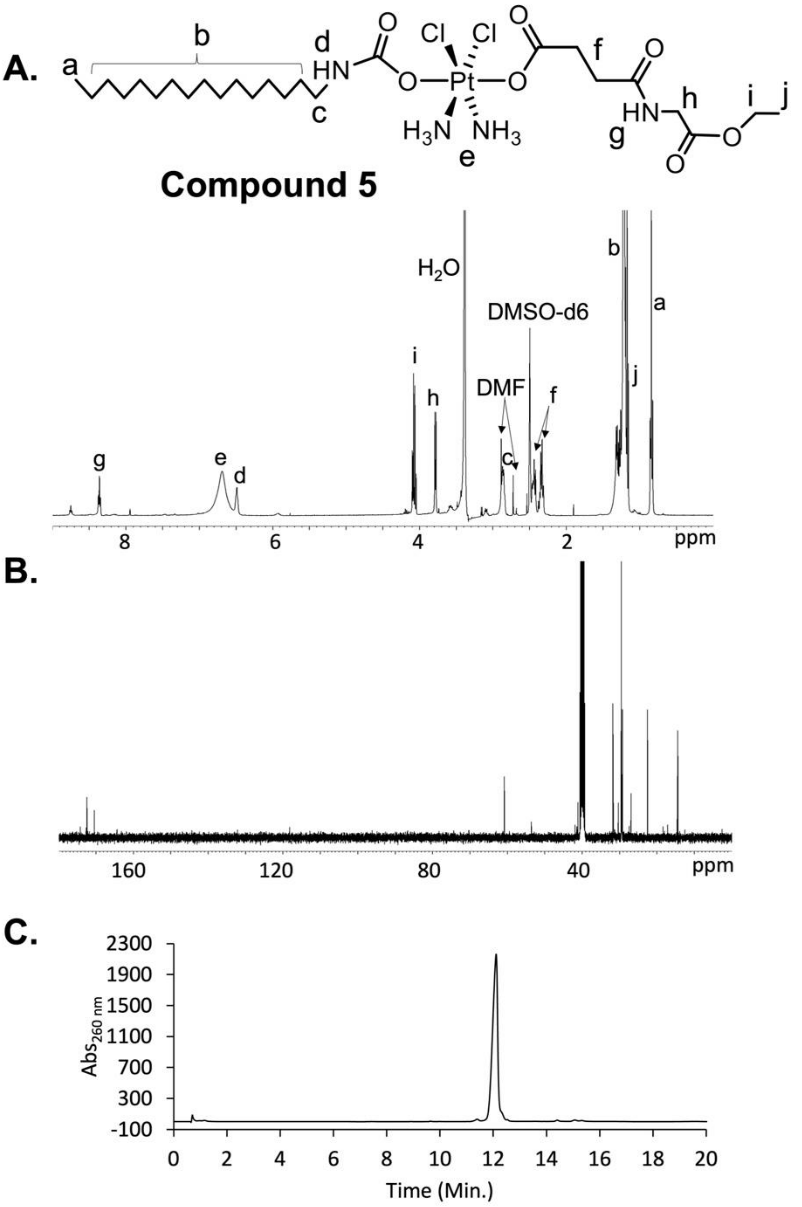

Figure S5. Characterization of Compound **5**: A. ^{1}H NMR; B. ^{13}C NMR; C. HPLC.

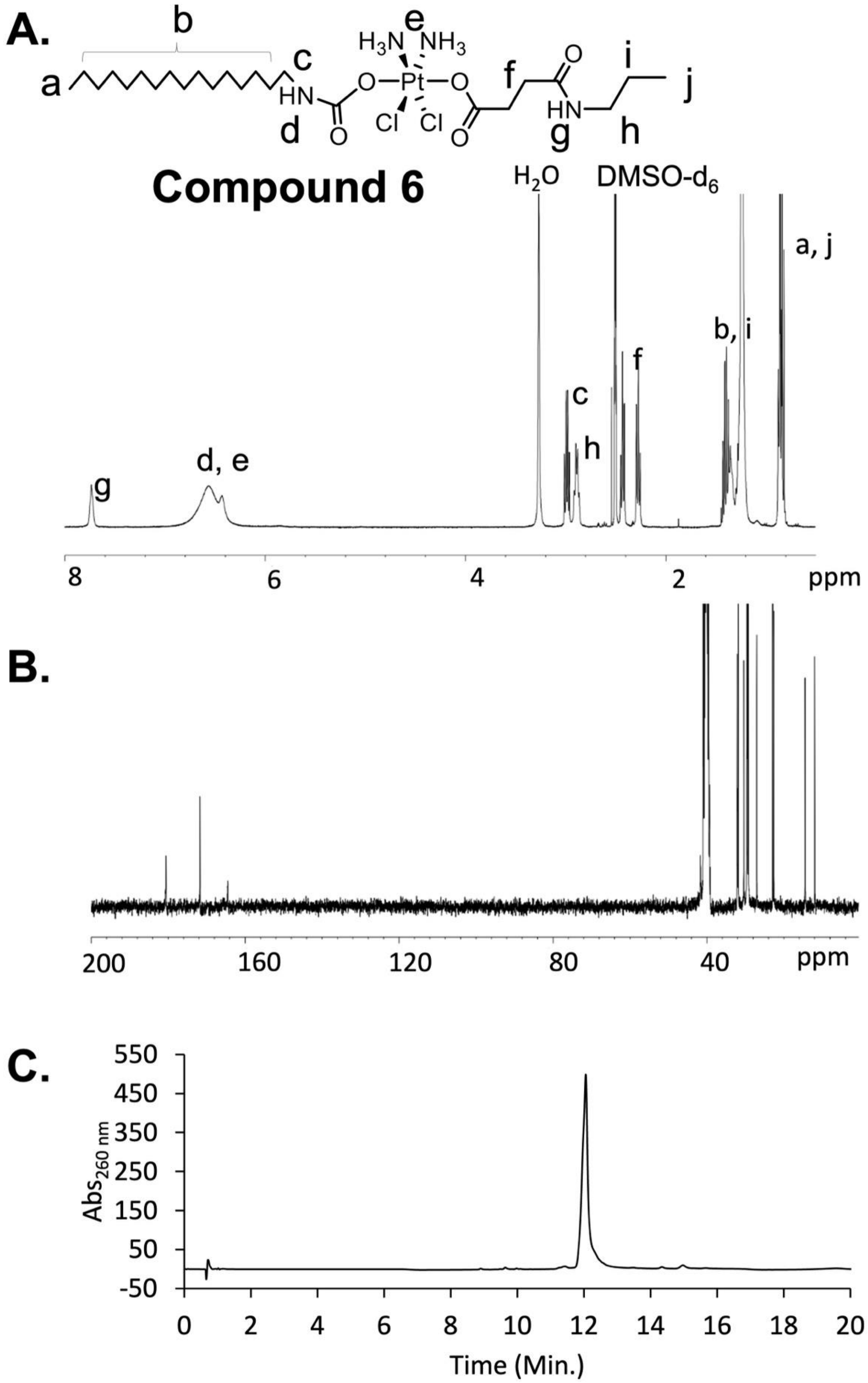

Figure S6. Characterization of Compound **6**: A. ^{1}H NMR; B. ^{13}C NMR; C. HPLC.

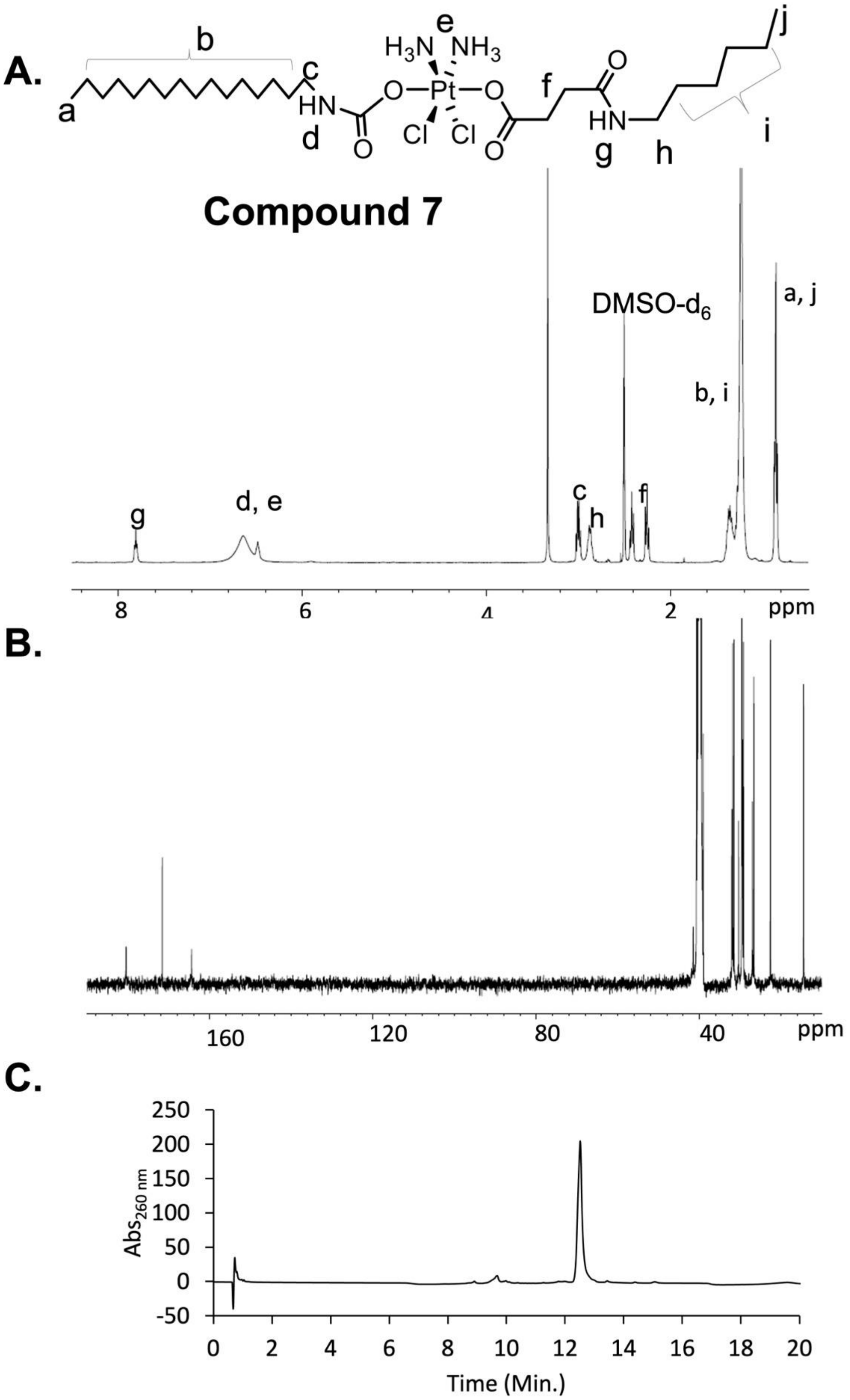

Figure S7. Characterization of Compound **7**: A. ^{1}H NMR; B. ^{13}C NMR; C. HPLC.

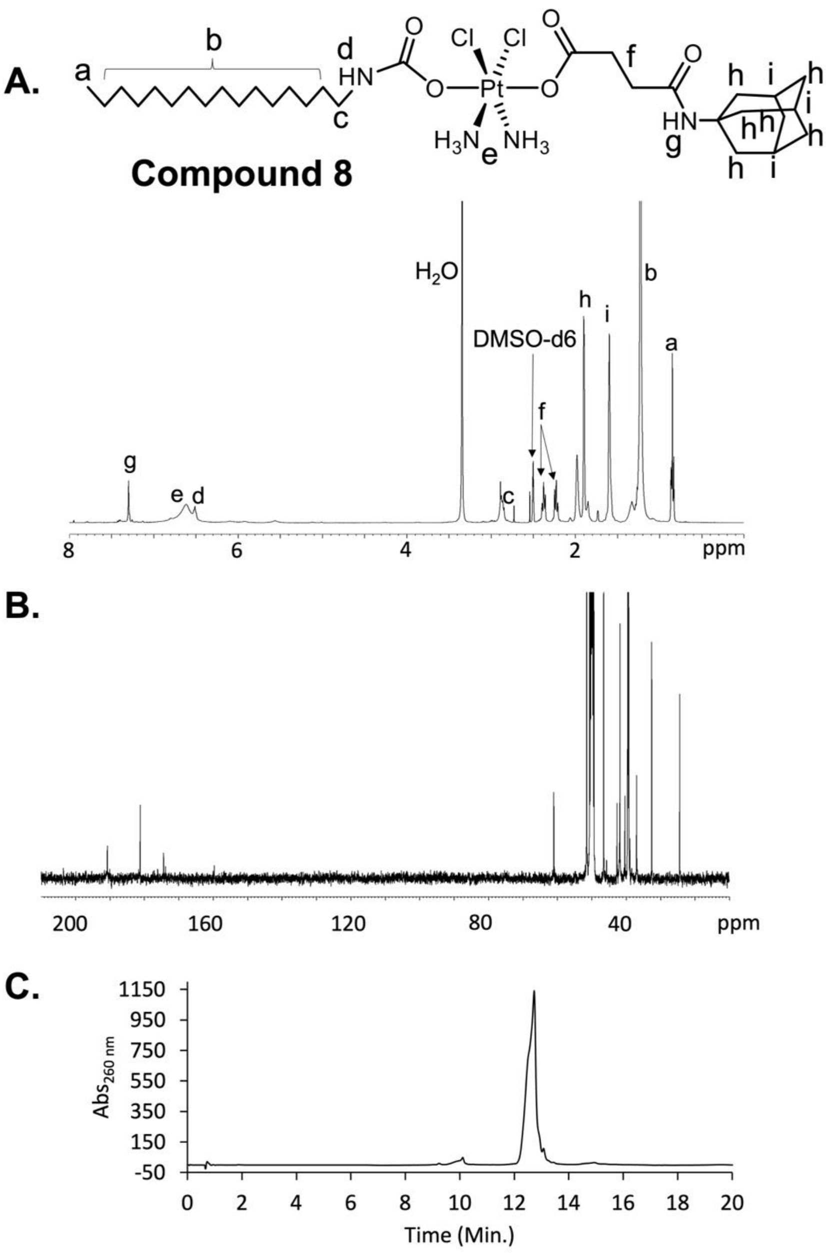

Figure S8. Characterization of Compound **8**: A. ^{1}H NMR; B. ^{13}C NMR; C. HPLC.

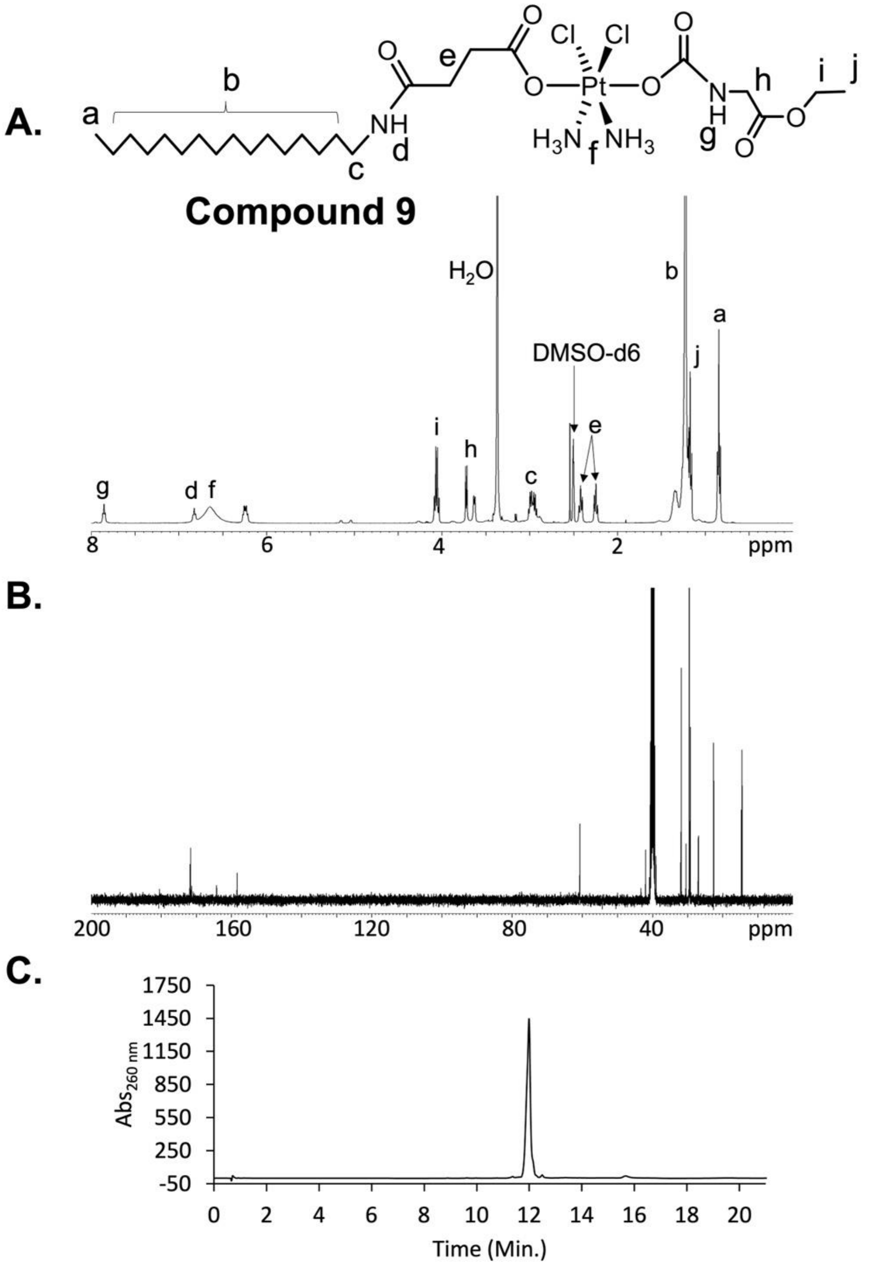

Figure S9. Characterization of Compound **9**: A. ^{1}H NMR; B. ^{13}C NMR; C. HPLC.
